# An exploratory analysis of the nature of informal knowledge underlying theories of planned action used for public health oriented knowledge translation

**DOI:** 10.1186/s13104-015-1391-6

**Published:** 2015-09-09

**Authors:** Anita Kothari, Jennifer A. Boyko, Andrea Campbell-Davison

**Affiliations:** School of Health Studies, Western University, Labatt Health Sciences Building, Room 222, London, ON N6A 5B9 Canada; School of Health Studies, Faculty of Health Sciences, and Faculty of Information and Media Studies, Western University, Labatt Health Sciences Building, Room 403, London, ON N6A 5B9 Canada; Faculty of Information and Media Studies, Western University, 1151 Richmond St., London, ON N6A 3K7 Canada

**Keywords:** Knowledge translation, Informal knowledge, Public health, Theories of change, Secondary analysis

## Abstract

**Background:**

Informal knowledge is used in public health practice to make sense of research findings. Although knowledge translation theories highlight the importance of informal knowledge, it is not clear to what extent the same literature provides guidance in terms of how to use it in practice. The objective of this study was to address this gap by exploring what planned action theories suggest in terms of using three types of informal knowledge: local, experiential and expert. We carried out an exploratory secondary analysis of the planned action theories that informed the development of a popular knowledge translation theory. Our sample included twenty-nine (n = 29) papers. We extracted information from these papers about sources of and guidance for using informal knowledge, and then carried out a thematic analysis.

**Results:**

We found that theories of planned action provide guidance (including sources of, methods for identifying, and suggestions for use) for using local, experiential and expert knowledge.

**Conclusion:**

This study builds on previous knowledge translation related work to provide insight into the practical use of informal knowledge. Public health practitioners can refer to the guidance summarized in this paper to inform their decision-making. Further research about how to use informal knowledge in public health practice is needed given the value being accorded to using informal knowledge in public health decision-making processes.

**Electronic supplementary material:**

The online version of this article (doi:10.1186/s13104-015-1391-6) contains supplementary material, which is available to authorized users.

## Background

A general dissatisfaction is emerging with current models of evidence-based practice that emphasize research evidence at the expense of other types of knowledge [1–3]. A public health manager or policymaker may need to make decisions about topics for which insufficient research evidence is available to direct change. In addition, research about the effectiveness of interventions might not be available due to the context-sensitive nature of community-based health promotion interventions. For example, there is a lack of high quality evidence for policy interventions to decrease health inequities and the evidence that does exist must consider local population-based trends related to inequalities (e.g., socioeconomic status) [4]. Nevertheless, public health practitioners are expected to incorporate evidence in their decision-making. For the most part this evidence is conceptualized as synthesized research findings.

There is an important growing literature demonstrating the role of informal knowledge in public health program planning. Kamper-Jõrgenson [5] argues that evidence-based public health integrates formal knowledge (i.e., arising from research) and informal knowledge (i.e., arising from experience or training). Informal knowledge draws from informal learning and may or may not be captured in written form. For example, a public health nurse assigned to an inner city homelessness initiative will learn valuable contextual knowledge when s/he begins working in the community with those in need of housing support. In contrast, formal knowledge comes from formal learning processes (e.g., application of the scientific method) and is often captured in written form (e.g., published peer-reviewed papers, or grey literature). We recognize the knowledge is not easily categorized as either formal or informal. For example, a database maintained by a local public health unit that includes “counts” of birds tested positive for the West Nile virus would be considered local, but a national data set developed and maintained to systematically track this information for the purposes of surveillance monitoring would be considered formal. The difference (in keeping with our definition) being whether the database was developed through systematic means and informed by science. According to the Organisation for Economic Co-operation and Development (OECD) [6], informal learning can be considered learning by experience or doing and can be characterized by a lack of formal learning objectives and intention to learn. Such informal learning might take place when a community health worker runs a collective kitchen for the first time. Public health professionals have described their preferred sources of informal knowledge as being drawn from client circumstances and preferences, personal experiences, and colleagues [7, 8]. Informal knowledge can be used to situate research findings in the local community context [8, 9] or more generally to make sense of data [10].

It is important to recognize that while the public health field is placing greater emphasis on understanding the sources of knowledge used in evidence-informed decision-making, other fields of inquiry already have an advanced understanding of knowledge types and practice-relevant applications of them. For example, management and organizational behaviour theorists describe tacit knowledge as “know-how” gained from informal learning processes [11, 12] that is used for non- market oriented tasks such as bartering and trading [11]. A review from the educational psychology literature provides a framework of types and qualities of knowledge that can be used for assessing learning goals or instructional goal [13], demonstrating how far along this area has come with respect to identifying and applying knowledge types. Organisations but may have little meaning for them and not be readily actionable. Many other fields, such as sociology [14], have also approached the problem of understanding informal knowledge. For this study, we borrow common concepts like experiential knowledge to think about and sometimes describe this study but public health’s unique characteristics [15] requires a context-sensitive approach to the application of knowledge types.

The emerging public health literature related to informal knowledge reflects various types. One type is considered to be local knowledge or that which comes from individuals in their specific social contexts and is shaped by (among other factors) gender, ethnic background, personal history, and community understanding [16]. Other research distinguishes the explicit, formalized or codified knowledge possessed by practitioners from the informal knowledge practitioners’ gain from experience learning in their own professional context [17, 18]. Informal knowledge can also take the form of knowledge held by scientific or professional experts in a particular field [12]. Experts gain knowledge formally (e.g., post-secondary training) and informally (e.g., practice and working with specific high-need populations). There is a well-established scientific literature base about experts and how they may differ from non-experts in terms of their development, training, reasoning, knowledge, social support, and innate talent [19–22]. Despite the various types of informal knowledge that are apparent from the literature, there is no agreed upon framework for identifying and defining them.

While some well-accepted knowledge translation (KT) frameworks do point to the importance of clinical expertise, patient preferences, local data, and context [23, 24], little direction exists in the published literature for public health practitioners in terms of using informal knowledge. In one study, the validity of informal knowledge was determined by personal knowledge, intuition, and the level of trust and experience held by the person conveying the knowledge [8]. Issues related to timeliness and relevance may also be relevant to supporting the use of informal knowledge [9].

The published KT literature does include guidance on how to use research evidence in decision-making [23–27]. A commonly referred to model among public health practitioners is the Knowledge-to-Action Framework [23], which is based on theories of planned action. According to Graham and Tetroe [28] planned action or change refers to logically and systematically engineering change to occur in a specific group or setting. In this regard, a planned action theory could be used to devise an intervention to improve evidence-informed decision-making. Planned action theories can also be considered collectively to inform the theoretical development of models or frameworks for use in specific contexts (e.g., as a tool to support public health planners).

Given the importance of informal knowledge in public health practice and the lack of guidance on how to use it, we wanted to explore the nature of informal knowledge underpinning KT practice in public health. Since Graham’s Knowledge-to-Action Framework [23] is commonly applied within public health practice [29], we focused our study on analysing the planned action theories comprising its theoretical basis. Further, development of the KTA framework did not include an assessment of the nature of the knowledge used by planned action theories, not is such an assessment available from the within the public health oriented KT literature.

The question that guided our study was—what do the planned action theories on which the KTA framework is built suggest in terms of using three specific types of informal knowledge (local, experiential, and expert) in practice? We defined local, experiential, and expert knowledge as follows: (a) local knowledge is that which is acquired from local sources of information (e.g., individuals, groups, organizations) and is specific to the local context (e.g., the number of children in the city who received their flu shot last year); (b) experiential knowledge is that which builds on formal and specialized education and training, but is developed through experience in a particular field of practice over time (e.g., working as part of a flu vaccination team for inner city schools); and (c) expert knowledge is that which is acquired from formal and specialized education and training and that is specific to a profession/occupation/vocation (e.g., how to give an injection). Salient differences between these types of knowledge (as we have defined them) are how/where the knowledge was acquired and what the knowledge pertains to. However, these types of knowledge also differ (albeit less clearly and with more overlap) in terms of how each is utilized in decision-making processes. We devised these definitions based on our understanding of the relevant theoretical literature about knowledge types in the public health field. Further, as knowledge-related terms have been offered by many authors and now carry a variety of meanings across a vast body of literature [30], these definitions are intended to operationalize our study and ensure our interpretations are meaningful to public health oriented KT researchers and practitioners.

## Methods

We carried out an exploratory secondary analysis of a select group of studies with respect to the use of local, experiential, and expert knowledge. We used an existing dataset (i.e., the planned action theories on which the KTA framework) in order to build on previous KT-related work and provide insight into the practical use of informal knowledge that can be the basis for more in-depth study. Exploratory research is useful when seeking to gather preliminary information about a topic in order to lay some groundwork for future research, while descriptive and research aims to explore and explain by producing this descriptions that move understanding of a topic forward [31].

### Data collection and sample

This study was an exploratory, secondary data analysis of the theoretical papers (n = 31) from which a commonly used knowledge-to-action model [23] was built. This framework has been accepted as the predominant KT framework by the Canadian Institutes for Health Research and other leading institutions. A review of more than thirty planned action theories led to the development of this framework, which encompasses both research and other forms of knowledge [28]. Given the prominence of this framework in the KT field, we purposively returned to its primary articles to explore what the planned action theories suggested in terms of using informal knowledge.

### Data extraction and analysis

We developed a structured data extraction form to systematically collect data about each theory. The extraction form focussed on the types of informal knowledge sources described in each paper and the guidance provided for these sources. The definitions of local, experiential, and expert knowledge discussed previously informed the data extraction process. More specifically, we extracted data related to theory properties (i.e., purpose, intended field of application, underlying principles/philosophies, key concepts/constructs, as well as how local, experiential, and expert knowledge was characterized (sources, methods for identifying, suggestions for use, and detailed instructions)). Excel was used to compile the extracted data into textual descriptions. One team member primarily carried out the data extraction and then compiled it. However, data from a subset of the papers (n = 10) were extracted by a second team member. Discrepancies were discussed among the two researchers until consensus was reached (although very few discrepancies occurred). The few discrepancies that occurred related to the categorization of types of knowledge given that there is some conceptual overlap between the definitions of local, experiential and expert knowledge we used. When all the data had been compiled, approximately five meetings were held with the three member research team to discuss the findings. This process involved collectively discussing, interpreting, and clarifying the data, as well as resolving any conflicts related to the data in order to produce a coherent summary [32] of the overall data.

## Results

Twenty-nine (n = 29) of the papers about planned action theories on which the Knowledge-to-Action Framework [23] was based were located and retrieved (see Additional file 1: Table S1). Among these papers, six were informative (i.e., described how planned action works) and 22 were directive (i.e., described how to do it). We were unable to locate two sources [33, 34]. One of these sources was not available to us at the time of this study and the other source could not be obtained from the authors. A summary of the data we compiled from the individual papers (and corresponding theories) is presented below.

### Using local knowledge

Seventeen out of the 29 papers suggested using local knowledge to plan change efforts (Additional file 1: Table S2). These papers identified various sources of local knowledge including intervention groups, key stakeholders, and staff (Additional file 1: Table S2: 1–10; 12–17). Informal knowledge could also be obtained from: the organizational context, current practices, benchmarking databases, and epidemiological data (Additional file 1: Table S2: 2; 4; 8; 9; 11–13; 15–17).

Methods for collecting local knowledge included: using focus groups (Additional file 1: Table S2: 1–4; 7–14; 16; 17), surveys for collecting individually-focused knowledge (Additional file 1: Table S2: 1; 3–5; 8; 13; 14; 16; 17) and other methods such as checklists, direct observations, chart audits, and performance reports (Additional file 1: Table S2: 1; 4; 6–8; 15–17). Local knowledge could also be found through needs assessments (Additional file 1: Table S2: 1; 13) or pilot testing of interventions (Additional file 1: Table S2: 1; 9; 16).

Of the papers mentioning local knowledge, 14 provided suggestions about when to use it during the planned action process (Additional file 1: Table S2: 1; 3; 4; 6; 8–17). For example, suggestions were provided such as using local knowledge to establish baseline measures and identify barriers and incentives (Additional file 1: Table S2: 1; 4; 6; 8; 9; 12; 13), as well as to tailor the intervention (Additional file 1: Table S2: 1; 7; 9; 12). It was also suggested that during the planned action process local knowledge could be used to monitor implementation processes (Additional file 1: Table S2: 7) and near the end of the change effort local knowledge could be used to evaluate outcomes (Additional file 1: Table S2: 1; 4; 9; 11–14; 16), interpret findings (Additional file 1: Table S2: 1; 11; 17) or as a way to work with stakeholders (Additional file 1: Table S2: 7; 11).

Seven papers provided explicit tools or detailed guidance on how to use local knowledge. Tools included such things as matrices (Additional file 1: Table S2: 1) and worksheets (Additional file 1: Table S2: 3) and could be used to assess readiness and need (Additional file 1: Table S2: 13) or for determining how to present information (Additional file 1: Table S2: 17). Detailed instructions ranged from when and how to evaluate change and make decisions to adapt, adopt, or reject (Additional file 1: Table S2: 16) to using an implementation team to categorize stakeholders (Additional file 1: Table S2: 4). Although detailed guidance on how to use local knowledge was identified from the papers, we did not identify this type of data in relation to experiential and expert knowledge.

### Using experiential knowledge

Twenty-seven papers suggested using experiential knowledge in planned action initiatives (Additional file 1: Table S3). Sources for experiential knowledge came from experienced individuals such as opinion leaders and mentors (Additional file 1: Table S3: 1; 3; 5–8; 11; 13; 16; 17; 19; 22; 26; 27), leaders or decision-makers (Additional file 1: Table S3: 1–3; 5; 9; 10; 15; 16; 17; 19; 20; 22; 23; 24; 27), colleagues (Additional file 1: Table S3: 2–9; 12; 14–16; 19-22; 24–26; 27), and stakeholders (Additional file 1: Table S3: 2; 6; 7; 11; 13; 15; 19–21; 26).

Further guidance was provided in 11 papers regarding the specific characteristics and skills individuals should have in order to be considered experiential knowledge sources. To illustrate, such individuals should be leaders (Additional file 1: Table S3: 1; 18; 20; 22), motivating (Additional file 1: Table S3: 18; 20), trustworthy (Additional file 1: Table S3: 6; 17; 20), and committed (Additional file 1: Table S3: 3; 6; 20; 22). Some theories also provided specific ways by which to identify experiential knowledge sources such as using a questionnaire, observation, interview or self-reports (Additional file 1: Table S3: 5), turning to a particular role such as a health educator (Additional file 1: Table S3: 2; 14) or using a committee (Additional file 1: Table S3: 3; 6).

### Using expert knowledge

Professional expert knowledge was included in 17 of the papers about planned action theories (Additional file 1: Table S4). In some cases, the use of experts was suggested without further elaboration about whom this might be (Additional file 1: Table S4: 2; 9; 10; 13; 15). However, when specified, experts could be researchers (Additional file 1: Table S4: 1; 8; 12; 14), nurses (Additional file 1: Table S4: 4; 6; 16; 17), educators (Additional file 1: Table S4: 7; 8), teams (Additional file 1: Table S4: 2; 3) or other specialized individuals, such as knowledge brokers (Additional file 1: Table S4: 11) or librarians (Additional file 1: Table S4: 17).

Theories suggested using the experts in different ways. Researchers, for instance, could be used as a resource to help identify and recommend effective interventions, develop and disseminate intervention packages, clarify interventions, note fit of planned action intervention to target group, help develop evaluation instrument/measures, and demonstrate the value of the intervention (Additional file 1: Table S4: 1; 8; 12; 14). As another example, nurses could take on different responsibilities depending on speciality. For example, clinical nurse specialists could evaluate readiness, establish clinical problem identification, develop innovation with staff or establish rapport with key leadership people (Additional file 1: Table S4: 6); advanced practice nurses could lead teams and suggest ways to read and synthesize research (Additional file 1: Table S4: 16); and comfort therapy service nurses could follow patients and provide educational materials (Additional file 1: Table S4: 17).

## Discussion

This exploratory analysis of the planned action theories underpinning the KTA framework provides a starting point for learning more about how to use informal knowledge in public health practice. The main finding to emerge from our study is that it appears our understanding of how to locate and use local knowledge is more advanced than our understanding of experiential and expert knowledge despite the availability of a well-developed research base. Overall, the papers revealed that local knowledge can be derived from a range of sources and that there are diverse ways to use these sources, such as using them at different stages in a KT planning initiative. Of the three knowledge types, the act of finding or using local knowledge sources was the most concrete and theories provided many examples of specific tools (e.g., checklists) and detailed guidance. It is likely that approaches developed for other research purposes are easily transferred into this domain. In other words, traditional research approaches like needs assessments and evaluations also capture local knowledge [35]. As a result, local knowledge might be easier to identify and measure than experiential and expert knowledge, accounting for the relatively extensive number of tools found in this area. Raphael [36] argues that the importance of local knowledge is to gain a critical perspective from stakeholders to supplement research literature.

The idea of using an opinion leader, mentors or involving colleagues as a way to learn about experiential knowledge was a prominent theme. This might be due to the fact that the use of an opinion leader is a common way to stimulate change in multiple disciplines [37] or that the development of clinical practice guidelines has traditionally included expert opinion in the absence of sufficient empirical evidence [38]. Consequently, other ways to tap into experiential knowledge received minimal attention. Experiential knowledge might be seen as tacit-like, that is, deeply personal and tied to context and often taken for granted [39]. Research focussed on uncovering such knowledge is available from the health sciences literature (as well as more broadly from the cognitive science and educational psychology literature) [40–42]. Therefore, it was surprising that little guidance was found in this sample beyond the use of opinion leaders.

In our analysis we classified expert knowledge as a source when the individual in question would be accessed specifically for their professional training expertise (e.g., a librarian). However, theories did not always provide explicit directions regarding how to utilize expertise and those that did provided a range of recommendations rather than a standard approach. This might reflect a lack of consensus about how to utilize expert knowledge effectively or may be due to the varied roles that experts play. For example, although we know that the use of expert knowledge in public policymaking depends on the political context, we do not know how to effectively bring balance to expert knowledge and public opinion in democratic decision making [43]. It may also be that the theories we examined were not informed by the broader cognitive science literature, which includes many studies focussed on understanding how to develop or harness expert knowledge [19, 20, 44, 45].

Discussions regarding how to use non-research evidence forms are growing in the public health oriented KT policymaking literature. Health policy decisions must be supported by various forms of evidence including research, judgements, values, and other factors [46]. Informal knowledge sources are often necessary when linking research to policy in order to ensure that ideas, interests, institutions, and external events are appropriately incorporated into the evidence-informed policymaking processes. An example of a KT strategy currently being used to support policymaking is deliberative dialogues, which are a type of group process that can help to integrate and interpret scientific and contextual data for the purposes of informing policy development [47]. Another example are evidence briefs that systematically and transparently identify, select, appraise, and synthesize systematic reviews, research studies, and context-specific data in order to address all elements of a policy question [48].

The Canadian National Collaborating Center for Healthy Public Policy (NCCHPP) developed an approach to knowledge synthesis that incorporates the use of deliberative dialogues and evidence briefs [49]. The method consists of four steps that each incorporate the use of informal knowledge: (1) an initial exploration of organizational websites (grey literature) and systematic review websites (scientific literature) to define the health issue; (2) construction of a logic model (with input from experts) to guide the knowledge synthesis process; (3) a formal review of the scientific and grey literature; and (4) a deliberative dialogue (informed by consultations with experts, decision makers, and citizens) that involves bringing various actors together to review the evidence and use their experience to contextualize it [49]. Similar processes have been described when policymaking is complex [50] or when systematic reviews have to be locally adapted for policymaking [51]. These examples signal a growing recognition that alternative sources of evidence are required to achieve sound health policy.

Public health practice, like policymaking, is also a complex process. Often research about effective interventions is not available or applicable to the population at hand. This is an important reason why the concept of evidence-based practice includes the integration of research alongside with clinical expertise, patient values, and resources. Our analysis compels us to ask—how can strategies such as deliberative dialogues and evidence briefs that support the use of informal knowledge in decision-making be used in day–day decision-making to support public health practitioners?

The work presented here is exploratory. The findings from this study are limited in that our sample was drawn from the theories used in one knowledge-to-action model [23], thus we may have missed other relevant theories available in print and online. Furthermore, it was sometimes difficult to separate out experience from expertise as some of the theories were ambiguous and there was some conceptual overlap between the definitions of local, experiential and expert knowledge that we used. For example, individuals labeled as change agents and opinion leaders were often identified as having both experiential and expert knowledge. We systematically classified this advice as experiential when the texts were focused on their experience as opposed to any professional training. However, in doing so we may have misinterpreted the authors’ intentions. Knowledge brokers on the other hand were placed in the expert knowledge category as they were identified as experts with specific training. Although our methodology lacked a comprehensive search strategy and quality assessment of individual studies, the broad scope of the study allowed us to produce useful (albeit preliminary) insights about using informal knowledge in public health decision-making processes.

Despite these limitations, this review provides preliminary guidance for understanding public health practice oriented KT whereby informal knowledge is used to learn about and make decisions about the application of research evidence in specific situations. However, a comprehensive study of how to identify and use informal knowledge in public health decision-making processes is needed. Such a study should include a database search for the full range of models, frameworks, theories or other sources that provide direction in the use and identification of informal knowledge sources for practice. In addition, other areas for future research include determining what is good quality informal evidence given the emergence of grey literature databases; developing methods for synthesizing good quality informal evidence for reliable decision-making; and concurrently developing supports for public health departments and practitioners to use good quality informal evidence in decision-making.

## Conclusion

This paper represents progress in terms of understanding the use of informal knowledge in public health program planning. Our findings can be used by public health practitioners wishing to include forms of informal knowledge into the decision-making process. Pushing the KT field in this direction may be uncomfortable for those who suggest that ‘research evidence’ is the only knowledge upon which to develop programs and policies. We are reminded, however, not to confine ourselves to certain worldviews that impede new ways of doing and understanding [3]. Eventually, new conceptualizations of KT that give more prominence to the integration of research evidence with informal knowledge sources will need to demonstrate program, practice and policy improvements. An initial step towards realizing improvements should include in-depth empirical examination of the types of informal knowledge that public health practitioners use that is informed by the broader literature. In the meantime, further review of the literature and research in areas of informal knowledge identification and use in front line public health practice is warranted.

## Additional file

**Additional file 1.** **Table S1.** Summary of planned action theories as described in included papers. **Table S2.** Characterizations of local knowledge use. **Table S3.** Characterizations of experiential knowledge. **Table S4.** Characterizations of expert knowledge.
