# Supplementary material for: An exploratory analysis of the nature of informal knowledge underlying theories of planned action used for public health oriented knowledge translation
Source: BMC Res Notes. 2015 Sep 9;8:424. doi: 10.1186/s13104-015-1391-6 (PMC4565051; doi:10.1186/s13104-015-1391-6)
Supplement: Supplementary file 1 — Additional file 1. Table S1. Summary of planned action theories as described in included papers. Table S2. Characterizations of local knowledge use. Table S3. Characterizations of experiential knowledge. Table S4. Characterizations of expert knowledge. [file 13104_2015_1391_MOESM1_ESM.docx]

**SUPPLEMENTARY FILE**

**Table 1: Summary of planned action theories as described in included papers**

| **Paper** | **Theory Purpose^1^** | **Intended Field of Application** | **Key Elements** | **Underlying Models and Theories** |
| --- | --- | --- | --- | --- |
| 1. Ashford, J., Eccles, M., Bond, S., Hall, L. A., & Bond, J. (1999). Improving health care through professional behaviour change: introducing a framework for identifying behaviour change stragegies. *British Journal of Clinical Governance, 4*(1), 14-23. | Informational | Health Care | behaviour change strategies; a systematic approach; dealing with issues in a reflexive fashion | market segmentation, balance theory, congruity theory, cognitive dissonance theory, attribution theories, social learning theory, social exchange theory, social identity theory, diffusion of innovations |
| 1. Bartholomew, L. K., Parcel, G. S., Kok, G., & Gottlieb, N. H. (2001). *Intervention Mapping: Designing Theory and Evidence-Based Health Promotion Programs with PowerWeb*. London, England: MacGraw Hill. | Directive | Health Policy (information systems) | tailoring to local needs; theory based methods; practical strategies; clear objectives; stakeholder buy-in; evaluation | theory of planned behaviour, trans theoretical model, persuasion communication model, goal-setting theory, attribution theory, health belief model, self-regulatory theories, social cognitive theory, diffusion of innovations theory, social network and social support theories, stage theory of organizational change, organizational development theory, interorganizational relationship theory, agenda building theory, policy windows theory |
| 1. Benefield, L. E. (2003). Implementing evidence-based practice in home care. *Home Healthc Nurse, 21*(12), 804-809; quiz 810-801. | Directive | Health Care | interdisciplinary/inter-level partnerships; stages of readiness | framework of guiding principles for planning system change |
| 1. Craik, J., & Rappolt, S. (2003). Theory of research utilization enhancement: a model for occupational therapy. *Can J Occup Ther, 70*(5), 266-275. | Directive | Health Care (occupational therapy) | structured reflection; patient involvement; peer consultation | theoretical stages of research utilization, theory of research utilization enhancements in occupational therapy (TRUE-OT) |
| 1. Dearing, J. W. (2004). Improving the state of health programming by using diffusion theory. *J Health Commun, 9 Suppl 1*, 21-36. doi: 10.1080/10810730490271502 | Directive | Health Policy | integrated application of knowledge about attributes, opinion leadership, and clustering | diffusion theory |
| 1. DiCenso, A., Virani, T., Bajnok, I., Borycki, E., Davies, B., Graham, I., . . . Scott, J. (2002). A toolkit to facilitate the implementation of clinical practice guidelines in healthcare settings. *Hosp Q, 5*(3), 55-60. | Directive | Health Care | active and clear dissemination; systematic process; involvement of appropriate stakeholders; readiness assessment; resource availability; evaluation | none stated |
| 1. Dixon, D. R. (1999). The behavioral side of information technology. *Int J Med Inform, 56*(1-3), 117-123. | Informational | Health Care (and technology) | determination of fit via consideration of user knowledge and skills, as well as technological requirements; influence of perceived usefulness, ease of use, acceptance/adoption, available resources, sophistication, and capabilities | innovation diffusion theory, technology acceptance model |
| 1. Doyle, D. M., Dauterive, R., Chuang, K. H., & Ellrodt, A. G. (2001). Translating evidence into practice: pursuing perfection in pneumococcal vaccination in a rural community. Respir Care, 46(11), 1258-1272; discussion 1273-1255. | Directive | Health Care | clear understanding of the problem; a systematic approach; effective team leadership; a case for change; institutional buy-in; behavioural barriers and effective strategies to deal with these; progress evaluation; adjustments based on evaluation | behaviour change theory |
| 1. Feifer, C., & Ornstein, S. M. (2004). Strategies for increasing adherence to clinical guidelines and improving patient outcomes in small primary care practices. *Jt Comm J Qual Saf, 30*(8), 432-441. | Informational | Health Care | local adaptation; prioritizing involving all staff; redesigning delivery systems; involving patients; using information systems; external guidance; performance data | grounded theory, complexity theory, organizational change frameworks, chronic care model, practice innovation, patient activation |
| 1. Graham, I. D., & Logan, J. (2004). Innovations in knowledge transfer and continuity of care. *Can J Nurs Res, 36*(2), 89-103. | Directive | Health Care | consideration of central elements (evidence based innovation, potential adopters, the environment); assessing the innovation adopters and environment for fit; identifying a change agent; clarifying the innovation; monitoring; evaluation | theories of change, process of reflection |
| 1. Grol, R., & Grimshaw, J. (1999). Evidence-based implementation of evidence-based medicine. *Jt Comm J Qual Improv, 25*(10), 503-513. | Directive | Health Care | concrete and easily understandable change plan; analysis of the target setting and group; identification of obstacles to change; links to needs, facilitators, and obstacles; multiple strategies; evaluation | innovation, communication, health promotion, and social marketing theories; behaviourist, social influence, organizational, and coercive approaches |
| 1. Grol, R., & Wensing, M. (2004). What drives change? Barriers to and incentives for achieving evidence-based practice. Med J Aust, 180(6 Suppl), S57-60. | Directive | Health Care | promotion of awareness, interest and involvement; creation of understanding; insight into routines; positive attitudes about change; trying out change; confirming value of change; integration of new practice into routines and the organization | cognitive, educational, attitudinal, and motivational theories |
| 1. Herie, M., & Martin, G. W. (2002). Knowledge diffusion in social work: a new approach to bridging the gap. *Soc Work, 47*(1), 85-95. | Directive | Social Work | market and system opportunities; engaging the system; field testing; dissemination; follow-up; system feedback; ongoing consultation | innovation diffusion theory, social marketing |
| 1. Hickey, M. (1990). The role of the clinical nurse specialist in the research utilization process. *Clin Nurse Spec, 4*(2), 93-96. | Directive | Health Care | system readiness; process support; process evaluation and improvement; dissemination | The Linkage Model |
| 1. Hyde, P. S., Falls, K., Morris, J. A., & Schoenwald, S. K. (2003). Turning knowledge into practice. Boston: Boston: The Technical Assistance Collaborative Inc. | Directive | Health Care | knowledge of clients, staff, and organization; advisory group of staff and population represenatives; literature review; determination of practices most likely to succeed; review of administrative, financial, and human resources practices, policies, and procedures; an action plan; evaluation and monitoring | the technology of knowledge transfer and adoption of innovation; change management |
| 1. Kraft, J. M., Mezoff, J. S., Sogolow, E. D., Neumann, M. S., & Thomas, P. A. (2000). A technology transfer model for effective HIV/AIDS interventions: science and practice. *AIDS Educ Prev, 12*(5 Suppl), 7-20. | Directive | Health Care (information technology) | pre-implementation (identify local needs and interventions, assess fit, prepare the organization and staff); implementation (secure technical assistance and conduct process evaluations); evaluation and monitoring | innovation diffusion theory, technology transfer |
| 1. Lavis, J. N., Robertson, D., Woodside, J. M., McLeod, C. B., & Abelson, J. (2003). How can research organizations more effectively transfer research knowledge to decision makers? *Milbank Q, 81*(2), 221-248, 171-222. | Informational | Health Care | understanding the message (the what), the audience (to whom), and the messenger (by whom); active transfer strategies; supporting communications and infrastructure; implementation evaluation | none stated |
| 1. Lundquist, D. G. (2003). A Rich Vision of Technology Transfer Technology Value Management. *The Journal of Technology Transfer, 28*(3-4), 265-284. doi: 10.1023/A:1024949029313 | Informational | Business (information technology) | requires a paradigm shift; answers the questions of ‘why’ (reason for the transfer), ‘who’ (who will be doing the transfer), ‘where’ (the environment), ‘at what cost’ (justification for the transfer), ‘how’ (understanding the stakeholders, context), and ‘what’ (the technology) | rich visioning |
| 1. Motwani, J., Sower, V. E., & Brashier, L. W. (1996). Implementing TQM in the health care sector. *Health Care Manage Rev, 21*(1), 73-82. | Directive | Health Care | understanding of and commitment to TQM/CQI; initial quality assessment; education of key personnel; setting a plan; programming (training, surveying, benchmarking, forming teams and councils, establishing measures); implementing (ongoing education, rewards, and committees); evaluation (annual evaluations and redesign or revisions) | TQM/CQI |
| 1. Moulding, N. T., Silagy, C. A., & Weller, D. P. (1999). A framework for effective management of change in clinical practice: dissemination and implementation of clinical practice guidelines. Qual Health Care, 8(3), 177-183. | Directive | Health Care | pre-implementation assessment of readiness, barriers, and the level at which the interventions should be targeted; multifaceted interventions; appropriately targeted support strategies and evaluations | innovation diffusion theory, social influence theory, trans theoretical model of behaviour change, health education theory |
| 1. National Health and Medical Research Council. (2000). How to put the evidence into practice: implementation and dissemination strategies. Canberra, Australia. | Directive | Health Care | statement of purpose; expert team; key messages; suitability of the information; progress review; resource availability; evaluation | industry and management theory, diffusion of innovations theory, trans theoretical model |
| 1. Pape, T. M. (2003). Evidence-based nursing practice: to infinity and beyond. *J Contin Educ Nurs, 34*(4), 154-161; quiz 189-190. | Directive | Health Care (guidelines) | knowledge brokers; problem identification; team development; guideline development; input from stakeholders; staff support; minimizing opposing arguments and challenges; ongoing education, monitoring and evaluation | IOWA model, Stetler model, diffusion of innovations theory, ACE Star model |
| 1. Proctor, E. K. (2004). Leverage Points for the Implementation of Evidence-Based Practice. *Brief Treatment and Crisis Intervention, 4*(3), 227-242. | Directive | Health Care | attainment of four intermediate outcomes (access, adoption, implementation, and assessment); supportive research, training, and organizational infrastructures; access to evidence; user-friendly interventions and materials; training; clear and straightforward benefits; linkage to values and norms, administrative pressure, resources, supervisors’ and agency leaders' commitment, knowledge, and support; incentives, prompts, and reminders; evaluation | knowledge diffusion, innovation and quality improvement |
| 1. Roberts-Gray, C., & Gray, T. (1983). Implementing Innovations: A Model to Bridge the Gap Between Diffusion and Utilization. *Science Communication, 5*(2), 213-232. doi: 10.1177/107554708300500204 | Directive | Business | values, commitment, and practices of the user; characteristics of the innovation; application of strategies via the developer (assistance, power, education, and persuasion); stages (orientation stage, trial use stage, integration stage, and end when becomes routine); recurrent analysis of fit between innovation and user; adjustment as needed | programme implementation theory, Lewin's theory of social change |
| 1. Rosswurm, M. A., & Larrabee, J. H. (1999). A model for change to evidence-based practice. *Image J Nurs Sch, 31*(4), 317-322. | Directive | Health Care | Stakeholder involvement; collection of internal data and comparisons with external data; problem identification; standard classification systems and language; evidence search, critique and synthesis; assessment of feasibility and risk; designing and proposing the change; identifying resources; planning the process; defining the outcomes; evaluation (pilot and outcome); staff education; monitoring | research utilization, change theory |
| 1. Simmons, R., Brown, J., & Diaz, M. (2002). Facilitating large-scale transitions to quality of care: an idea whose time has come. *Stud Fam Plann, 33*(1), 61-75. | Informational | Health Policy | consideration and understanding of the environment (user and innovation); scaling up (transfer of small scale pilots to larger scale projects); awareness of available resource and user systems; participatory organization development | diffusion of innovations theory |
| 1. Stetler, C. B. (2001). Updating the Stetler Model of research utilization to facilitate evidence-based practice. *Nurs Outlook, 49*(6), 272-279. doi: 10.1067/mno.2001.120517 | Directive | Health Care | critical thinking and decision making steps; preparation (purpose and outcomes, affirming priority, considering influential factors, literature searches and selection of evidence); validation (utilization-focused critique and synopsis); evaluation and decision-making (determining fit, feasibility, substantiating evidence, considering current practice); application (confirm type, level and method of application); evaluation (identify goals and outcomes) | none stated |
| 1. Titler, M. G., Kleiber, C., Steelman, V. J., Rakel, B. A., Budreau, G., Everett, L. Q., . . . Goode, C. J. (2001). The Iowa Model of Evidence-Based Practice to Promote Quality Care. *Crit Care Nurs Clin North Am, 13*(4), 497-509. | Directive | Health Care | problem identification; determining the priority of the implementation; forming a team; assembling, critiquing and synthesizing the research; collecting baseline data; designing, implementing and piloting the project; selecting the outcomes; continuous evaluation and dissemination of the results | none stated |
| 1. Tracy, S., Dufault, M., Kogut, S., Martin, V., Rossi, S., & Willey-Temkin, C. (2006). Translating best practices in nondrug postoperative pain management. *Nurs Res, 55*(2 Suppl), S57-67. | Directive | Health Care | identification of a clinical problem; critique of the evidence; evaluation of the research relevance for practice; design of evidence-based best practice standards, policies or protocols; implementation and evaluation; deciding to adopt, alter or reject the standards; development of a way to disseminate and  extend the innovation to other appropriate settings | diffusion of innovations |

^1^Informational: Purpose of the paper was to provide information/suggestions not direct steps/processes/procedures

Directive: Purpose of the paper was to provide specific steps/processes/procedures to follow

**Table 2: Characterizations of local knowledge use**

| **Paper** | **Source(s)** | **Methods for identifying** | **Suggestions for use** | **Detailed instructions** |
| --- | --- | --- | --- | --- |
| 1. (Bartholomew et al., 2001) | Intervention group | Needs assessment (PRECEDE model), intervention group information/checklists; focus group; pilot testing; observation | Theories and methods for change need to be tailored to intervention group; should use pilot testing at various stages | Create matrices based on needs assessment (says what to include in matrices; incl. performance objectives, determinants, learning and change objectives); use focus groups to validate performance objectives; brainstorm determinants that may undermine implementation; use program evaluator to evaluate outcomes based on initial needs assessment |
| 1. (Benefield, 2003) | Current practice; staff teams (discipline specific and ~~interdisciplinary~~) | Program wide assessment; focus group | Suggests use of facilitator for focus group; determine, refine, and generate research reports based on program wide assessment; (some suggestion of further assessment with various groups e.g. clients, resources, etc.) | n/a |
| 1. (Dearing, 2004) | Change agency staff; priority population; networks of intermediaries (characteristics and reactions to pilot) | Pre- and post-test survey; Identify a population by delineating population parameters, limits and inclusions; consultation | In consultation with change agency staff, decision is made regarding focus | n/a |
| 1. (DiCenso et al., 2002) | Stakeholders;  organizational context;  outcomes/ indicators | Worksheet; snowball technique to identify stakeholders; implementation team to interview, survey, and do focus groups; questionnaire;  chart audit; environmental readiness assessment | Evaluation (outcome measures) should be planned before implementation (may need baseline data) | Implementation team to interview, survey, and do focus groups and use the data to categorize stakeholders; implementation team to use worksheet to answer questions about the environment; for evaluation: identify measures, people who will collect data, and get approval |
| 1. (Dixon, 1999) | End user | Kaplan evaluation strategy; questionnaire | n/a | n/a |
| 1. (Doyle et al., 2001) | Physicians; patients | Checklist | Multidisciplinary team to do inventory of barriers using checklist | n/a |
| 1. (Feifer & Ornstein, 2004) | Staff; patients; electronic medical records | Performance report;  improvement meetings;  feedback loops; | Suggests use of performance reports as poster motivators with star stickers (gained external accountability/ motivation by advertising their improvements to patients); must consider local context; info loops provide quality back up (staff point out overdue tests and overlooked values that need attention (some organizations needed permission to speak up to doctors this way)); suggests use of electronic medical records to merge personal lab results, send results to researchers, and access subgroup info |  |
| 1. (Graham & Logan, 2004) | Potential adopters; practice environment | Focus group; survey; adopters perceptions assessment;  practice environment assessment (suggests questions to ask for these assessments); environmental scan (incl. Chart audit) | Assessment of potential adopters perceptions of characteristics of the innovation and barriers to adoption and current practices and habits of the practice environment | Change agent to do barriers assessment with identification of possible supports and facilitators |
| 1. (Grol & Grimshaw, 1999) | Target setting; target group | Pilot testing;  interactive session (focus group) | Proposal should be tested in the proposed setting; analyze the target setting and target group and tailor interventions and identify obstacles to change (barriers and facilitators); use interactive session if desired to raise awareness, stimulate acceptance or market the innovation; intervention strategies should be based on barriers and facilitators; continuous evaluation of intervention |  |
| 1. (Herie & Martin, 2002) | Frontline counsellors; agency managers; government representatives; target systems and populations | Consultations and regional focus groups; field test | Year-long field testing with follow-up consultations regarding results and revisions based on these results | n/a |
| 1. (Hyde et al., 2003) | Practitioners/staff and patients/clients;  stakeholders; program/organization;  finances; policies/procedures; politics | Fidelity scale; resources to review local knowledge collection procedures and outcome reports; team meetings and consultations | Need to develop evaluation plan indicating outcomes you will measure, data you need to collect, and methods to collect and the frequency of collection; use team meetings to keep stakeholders informed, identify problems, and brainstorm; regular reporting necessary | n/a |
| 1. (Kraft et al., 2000) | Epidemiological data; priority population; organizational characteristics; process; outcome; cost | Consultation;  formative assessment | Use local information to determine fit and identify citizen and advocacy groups | Collection and review of process data; should evaluate cost |
| 1. (Moulding et al., 1999) | Practitioners; organizational characteristics; patients; other health professionals | Needs assessment; Cohen’s tool; change agent to do practitioner needs assessment (survey, interview, or group consultation) | Change agents must identify with concerns of clinicians, stage of readiness, nature of barriers, social influence, and environmental support is important; type of evaluation tool to assess implementation will depend on setting and implementation strategy used | Needs assessment and Cohen's tool used to assess readiness and need; practitioners needs assessment should be qualitative; strategies are selected and designed based on assessments |
| 1. (National Health and Medical Research Council, 2000) | Audience (stakeholders/key players); representatives/working party | Surveys, interviews, focus groups; some examples of evaluation methods (outcome measures) | Evaluation of strategy and outcome depends on the implementation strategies used | Find out how the audience would like information presented, can be informal asking representatives what the various groups think; find out what the barriers are and for whom, barriers need to be identified and described, range of options for eliciting barriers differing in resource implications thus the team needs to make this decision using their own judgment based on skills and resources available; suggest qualitative methods |
| 1. (Roberts-Gray & Gray, 1983) | User characteristics (organizational structures/facilities, policies/regulations; individual abilities/behaviours, attitudes/values) | Checklists | Identify fit, compare innovation with characteristics of the user (describes important factors of the user and innovation that influence successful implementation) |  |
| 1. (Rosswurm & Larrabee, 1999) | Staff; stakeholders; benchmarking databases; patients | Survey; focus group; pilot test; practitioners collect internal performance data; external benchmark data | Practitioners decide what to measure (quality improvement and staff opinion) and prepare the questionnaires based on this Says who should be involved, types of surveys you can use; compare what | Stakeholder participation is encouraged through the whole process; training is done regarding outcome measures; says when to evaluate change and how to assess the results; decision to adopt, adapt, or reject is based on change evaluation; compare internal data with external benchmark data |
| 1. (Tracy et al., 2006) | Nurses; patients; staff; agency values/standards | Stetler Tools;  Non-Drug Complementary Pain Interventions Survey;  Ferrell's standard of care audit instrument;  Miller Behavioural Style Scale; roundSF: Table discussions; unit level staff nurse council members do chart audit | Recommendations are generated from roundSF: Table discussions | Three month chart audit with unit level staff nurse council members, led by hospital's nurse researcher to identify common problematic areas which have a strong body of validated empirical evidence; discussions should be regarding applicability and usefulness to the situation, and jointly develop recommendations for development of best practice protocols; nurses and patients were asked to review Interventions survey for readability user friendliness and length; audit instrument conducted on patient charts; short form of Miller Behavioural Style Scale was used to determine patients preferred method for receiving information (information-coping style) |

**Table 3: Characterizations of experiential knowledge**

| **Paper** | **Source(s)** | **Method(s) for identifying** | **Suggestions for use** |
| --- | --- | --- | --- |
| 1. (Ashford et al., 1999) | Change agent; opinion leader | Influential people who know the target group | n/a |
| 1. (Bartholomew et al., 2001) | Change agent; members of user/resource groups; staff team | Health educator is to identify change agent | Brainstorming, provides question examples to consider; providing input regarding context of implementation to ensure fit (tailor to context); providing creative input into resulting program components and materials (how the message will be delivered); change agent reviews literature; change agent and staff team to develop matrices with health educator/program planner; providing input into factors that will influence implementation (barriers/facilitators) |
| 1. (Benefield, 2003) | Champion/mentor; agency leader; staff team | Management to find champion/mentor, then team to select champion; must have positive outlook, be encouraging to others; possibly an expert staff nurse, clinical specialist, or clinical supervisor | Agency leader to learn the language and literature, recommend topics, and share ideas with champion/mentor; identify need (gives specific questions to consider); staff team, champion/mentor, and agency leader are to review models, assess strengths and weaknesses, identify barriers and facilitators, assess outcomes, schedule meetings, track progress, create timelines; selects and circulates research reports; provide input (consulting) |
| 1. (Craik & Rappolt, 2003) | Peers (staff) |  | Consultation; gain insight |
| 1. (Dearing, 2004) | Opinion leaders; change agency staff | Identify opinion leader via sociometric questionnaires, participant observation, personal interviews, and/or self-report | Consultation, talk about best practices with potential adopters |
| 1. (DiCenso et al., 2002) | Stakeholders; implementation team (staff); program evaluation team (staff);local champion/opinion leader | For local champion/opinion leader select unit based nurses with high interest and clinical expertise and peer respect; for program evaluation team select existing committee or individuals with experience in program evaluation; team/group identifies stakeholders using snowball technique | Questions to consider for evaluation and appraisal; use worksheets to answer questions about the environment; implementation team consults with stakeholders, use stakeholder analysis to identify barriers and facilitators, identify unpublished CPGs to evaluate/appraise, determines strategies for engagement, and develops written action plan |
| 1. (Dixon, 1999) | End users; colleagues; opinion leaders | Experienced with technology | n/a |
| 1. (Doyle et al., 2001) | Multi-disciplinary team; opinion leaders | Credible leadership | Frame projects as non-punitive and as issues of systems failure not individual failure; address goals up front; quantify opportunity in terms meaningful to members of the organization; team to do inventory of barriers using checklist; decide which implementation and dissemination strategies to use based on barriers, assess barriers throughout and modify strategies accordingly |
| 1. (Feifer & Ornstein, 2004) | Project leaders; staff | n/a | Project leaders obtain buy in from key stakeholders, actively supervise staff roles, and motivate staff; staff reorganize systems, take on new roles in assisting with care and are given more responsibility for monitoring |
| 1. (Graham & Logan, 2004) | Change agents/facilitators | n/a | Planning and tailoring implementation interventions and monitoring their progress; monitoring via process evaluations |
| 1. (Grol & Grimshaw, 1999) | Opinion leaders; key persons | n/a | Need consensus from opinion leaders regarding proposal; key persons can help market or raise awareness |
| 1. (Grol & Wensing, 2004) | Colleagues | n/a | n/a |
| 1. (Herie & Martin, 2002) | Key stakeholders; opinion leaders; champions | n/a | n/a |
| 1. (Hickey, 1990) | Staff; resource utilization task force (staff nurses) | Clinical Nurse Specialist to recruit task force of nurses | n/a |
| 1. (Hyde et al., 2003) | Practitioners and consumers; organizational decision-makers (board and executive management); stakeholder groups; staff | n/a | Development of stakeholder group needs to occur early and they should participate and advise in all aspects of the process (lit review, decision-making, design, etc.); lists questions to consider in consultations with practitioners and consumers; lists questions for stakeholders to consider in determining outcome measure(s)involve stakeholders in making action implementation plan lists what to consider and what to include; staff meetings to discuss practice |
| 1. (Kraft et al., 2000) | Champion; colleagues; administrators and staff | champions need: negotiation and promoting skills, some may require training or other assistance | Colleagues can narrow down interventions or suggest based on what they have used; champion can mobilize support by facilitating staff participation, keeping staff focused, securing resources, providing evidence of intervention effectiveness, finding ways to reward staff, and negotiating trade-offs and compromises; administrator and staff consultations |
| 1. (Lavis et al., 2003) | Opinion leaders | Opinion leaders must be credible | n/a |
| 1. (Lundquist, 2003) | Change agent | Skills required for change-agent: vision, learning, leadership, strategy, motivation (sales), cooperation, communication, relationships, effective marketing skills | n/a |
| 1. (Moulding et al., 1999) | Change agent; staff/clinicians, practitioners, opinion leaders; patient groups | Change agents must identify with the concerns of the clinicians; use surveys, interviews, and group consultations to obtain experiential information on staff and patient groups | staff (clinicians) involved in guideline development; consult patient groups; |
| 1. (National Health and Medical Research Council, 2000) | Team; system leader; priority population/consumer groups | System leader (has enough authority to make change when required and overcome barriers when identified; have authority (formal or moral) in all areas that will be affected by change; should be a senior person; add someone with technical expertise in the subject matter who is enthusiastic about change, person who can assume day to day leadership of a project (someone with time and enthusiasm and enough influence to encourage action); trust, approachable, good listening skills, and competent | Team carries out all actions, team develops formal summary of the current situation using research evidence, is responsible for managing the implementation and dissemination strategies, decision making, developing objectives, finding out how various audiences need to be addressed through surveys, administration; all stakeholders (staff, consumer groups) are included in clinical guideline development |
| 1. (Pape, 2003) | Stakeholders/key individuals; evidence based practice team | n/a | Stakeholders: investigate the issue with the knowledge broker, team: review documentation, analyze reviews, and formulate guideline; determine criticisms, opposing arguments, and best responses |
| 1. (Proctor, 2004) | Supervisors; program leaders; senior staff; opinion leaders; change agents | Program leaders/senior staff/opinion leaders/change agents need to be 'groomed' to promote innovation, must show commitment, knowledge and support, skilled in using EBPs, supervisors should be visible and influential | Supervisors: help with evidence understanding, use and access; program leaders, senior staff, opinion leaders, change agents: identify problems, supports and pressure for change, must provide incentives and rewards, change agents provide training |
| 1. (Roberts-Gray & Gray, 1983) | Leader | n/a | Assistance (technical or fiscal), education, power (establish rules or sanctions), persuasion, develop appropriate utilization plans/make changes in role responsibilities and endorse a concept |
| 1. (Rosswurm & Larrabee, 1999) | Administrators; practitioners; other organizations; staff | n/a | Practitioners decide what to measure and prepare questionnaires based on this. Training is done regarding these measures and inter-rater reliability. Describes when to do surveys, how to assess the results (quality improvement and staff opinion) |
| 1. (Simmons et al., 2002) | Teams/consultants | n/a | Establish an egalitarian relationship with members of the organization; help organization members identify new opportunities and solve their own problems; facilitation; must be experienced trainers and familiar with the organization |
| 1. (Stetler, 2001) | Team; Stakeholders; opinion leaders | n/a |  |
| 1. (Tracy et al., 2006) | 12 member comfort therapy service (opinion leaders); staff and nursing administration | n/a | Comfort therapy service: trained regarding the protocols; staff and nursing administration: decide whether to adopt or reject (collaboratively) |

**Table 4: Characterizations of Expert knowledge**

| **Paper** | **Source(s)** | **Method(s) for identifying** | **Suggestions for use** |
| --- | --- | --- | --- |
| 1. (Bartholomew et al., 2001) | Health educator/planner (program deliverer, program planner); creative resources (e.g. graphic designer); resource individuals (e.g. researchers); program evaluators | n/a | Health educator/planner to do needs assessment, identify ecological context, health related behaviours, and environmental conditions of at risk group, identify and write performance objectives (detailed description of behavioral objectives, etc. broken down into manageable component parts, very specific direction), Intervention mapping (intervention program planning), validate performance objectives (questioning and observing members of intervention groups and service providers); brainstorm, provide incentives, facilitate change; ask questions in the community, come up with detailed summary plan while considering all aspects (context, interface, determinants, etc.); creative resources should be hired when the health educator does not have the needed skill (e.g. graphic designer), gives direction on how to find creative resource and what and how to provide them with the information to do their job; program evaluator evaluates the intervention, development of the evaluation plan begins with needs assessment (very specific direction provided in this model) |
| 1. (Dearing, 2004) | Panel of experts | n/a | Panel of experts convened to rate programs, highest rated programs would constitute sample of best practices |
| 1. (DiCenso et al., 2002) | Implementation team; project management team member | n/a | Develop a group of 4 members (implementation team) who have expertise or experience in the clinical topic area of interest to evaluate/appraise; questions to consider; project management team member to use their project planning resources to make a detailed plan of each step in implementation and plan budget, tailor strategies to overcome barriers and build on the facilitators; specific direction given (e.g. Use results of env scan and stakeholder analysis to identify barriers and facilitators, enlist local champions, select strategies to make use of resources and supports on hand, etc.) |
| 1. (Feifer & Ornstein, 2004) | Staff (e.g. nurses, nurse practitioners or physician assistants) | n/a | Applications of expert knowledge: nurses given more responsibility for monitoring tasks; other examples of nurse practitioners or physician's assistants taking on different responsibilities |
| 1. (Grol & Grimshaw, 1999) | Experts; expert outreach visitors | n/a | Need consensus from experts regarding proposal; expert outreach visitors to convince target of innovations value |
| 1. (Hickey, 1990) | Clinical Nurse Specialist (CNS) | n/a | CNS to evaluate readiness, recruit task force, establish how clinical problems are to be identified, determine the process for procuring the research findings and analyzing them, and identifying existing incentives and barriers and establish plans to enhance or thwart them; develop innovation for implementation with staff, need to develop rapport with key leadership people, convince staff about need for research utilization, alleviate staffs concerns by providing information, holding meetings, using task force, coordinating classes, can take on facilitator or role model role; identify evaluation variables and people responsible for evaluation and revise if necessary |
| 1. (Hyde et al., 2003) | Experts; trainers; consultant in using fidelity scores | n/a | Talk with experts to find out what practices are out there; trainers to educate staff; consultant in using fidelity scores to measure fidelity |
| 1. (Kraft et al., 2000) | Prevention service providers, linking agents, researchers; technology assistance providers (in-house evaluators, behavioural and social scientists); trainers | Technology assistance providers: can use in-house evaluators, local college behavioural/social scientists, regional prevention centers, Centers for Disease Control and Prevention volunteers | [Prevention service providers might engage community groups, organizations, and members of the priority population by asking for their input throughout the process; identify target group needs, find (some info provided on where they can find interventions), assess fit (suggests things to consider), and tailor intervention with linking agents, researchers, and target group; work with technology assistance providers; conduct process evaluation] [linking agents (mediators) facilitate technology transfer by helping to translate and disseminate information, working with providers to tailor interventions and identify technology assistance needs, and attempt to bridge the communication gap between researchers and providers; help establish evaluation criteria] [researchers may identify effective interventions and develop and disseminate intervention packages to help providers in decision making, help develop evaluation instrument (some suggestion on what to include)] [trainers provide training (offers some suggestion on what to include)] [technology assistance providers may implement organizational changes that support the intervention, build capacity to plan for and use intervention; provide continued support and training; help develop evaluation instrument (some suggestion on what to include)] |
| 1. (Lavis et al., 2003) | Experts | Highly credible individuals | Can be used to deliver actionable messages |
| 1. (Motwani et al., 1996) | Specialists | n/a | Hired when required to help the implementation process |
| 1. (Pape, 2003) | Knowledge broker | n/a | Investigates the issue with key stakeholders; search internet and other sources for systematic reviews; if needed, policy would be redesigned in collaboration with knowledge broker |
| 1. (Proctor, 2004) | Researchers | n/a | Provide clear concise and detailed recommendations (reviews, manuals, guidelines); can enhance perceived simplicity of implementation by describing components clearly and in detail; can also note similarities; should be similar to the adopting culture |
| 1. (Roberts-Gray & Gray, 1983) | Expert | n/a | Expert is developer; assistance (technical or fiscal), education, power (establish rules or sanctions), persuasion; develop appropriate utilization plans/make changes in role responsibilities, provides user with info on benefits and demands associated with innovation; exercise rational-legal power to obtain commitment (letter of agreement, formal instruction); give access to instructional self-help material to facilitate own experiential learning (coaching) |
| 1. (Simmons et al., 2002) | Researchers | n/a | Are a resource system; use of policy windows (i.e. when an election of officials are committed to improving public health services) to draw attention to value of the innovation |
| 1. (Stetler, 2001) | Expert | n/a | Expert could synthesize research findings mentally |
| 1. (Titler et al., 2001) | Nurses; experts; advanced practice nurse | n/a | Talks about specific questions that should be asked to determine if it is relevant to the needs of the particular department, if it fits with priorities and if it excites staff (also a list of considerations when selecting a topic); leader of a team, suggests ways to delegate how to group articles, directs reader to aids, lists methods (journal club, assigning to students, etc.); list of factors for inclusion considerations and suggests methods (systematic review and meta-analysis) to synthesize research |
| 1. (Tracy et al., 2006) | Reference librarian; comfort therapy service nurse | n/a | Reference librarian assists in literature search and instruction; comfort therapy service nurse to follow patients, provide educational materials, do assessments throughout the hospital stay. |
